# Accuracy of climate-based forecasts of pathogen spread

**DOI:** 10.1098/rsos.160975

**Published:** 2017-03-29

**Authors:** Annakate M. Schatz, Andrew M. Kramer, John M. Drake

**Affiliations:** 1 Odum School of Ecology, University of Georgia, 140 East Green Street, Athens, GA 30602, USA; 2 Center for the Ecology of Infectious Diseases, University of Georgia, 140 East Green Street, Athens, GA 30602, USA

**Keywords:** species distribution model, *Batrachochytrium dendrobatidis*, machine learning, hindcasting

## Abstract

Species distribution models (SDMs) are a tool for predicting the eventual geographical range of an emerging pathogen. Most SDMs, however, rely on an assumption of equilibrium with the environment, which an emerging pathogen, by definition, has not reached. To determine if some SDM approaches work better than others for modelling the spread of emerging, non-equilibrium pathogens, we studied time-sensitive predictive performance of SDMs for *Batrachochytrium dendrobatidis*, a devastating infectious fungus of amphibians, using multiple methods trained on time-incremented subsets of the available data. We split our data into timeline-based training and testing sets, and evaluated models on each set using standard performance criteria, including AUC, kappa, false negative rate and the Boyce index. Of eight models examined, we found that boosted regression trees and random forests performed best, closely followed by MaxEnt. As expected, predictive performance generally improved with the length of time series used for model training. These results provide information on how quickly the potential extent of an emerging disease may be determined, and identify which modelling frameworks are likely to provide useful information during the early phases of pathogen expansion.

## 1. Introduction

Species distribution models (SDMs) are commonly used to predict the geographical range a species can occupy, based on environmental factors at locations where the species has been collected [1–3]. These predictions are valuable in many contexts, such as deciding where to sample for a species or assessing a species' potential to expand its range [2–4]. The need to address the impacts of climate change and invasive species has resulted in regular application of these models to geographical areas outside the region used for model fitting, to make predictions about where else a species might be found or where it could establish if introduced [1,5].

Typically, SDMs assume that the occurrence points on which they are based are independently and identically distributed with respect to the full range of the species' environmental tolerance, i.e. that the species has reached equilibrium with its environment [4,5]. SDMs' reliance on an assumption of equilibrium is a challenge for researchers investigating invasive species [5,6]. Knowing the implications of violating this assumption is a particularly serious concern when forecasting the niche of destructive pathogens, such as *Batrachochytrium dendrobatidis* (*Bd*). Newly discovered or emerging diseases are not likely to have reached equilibrium. Often, the most urgent question is where they will spread next.

Previous research on modelling *Bd* spread has attempted to predict its potential geographical range, globally or across a limited area, using random divisions of data into training and testing sets [7–11]. Research on other species has explicitly tried to adjust for the use of non-equilibrium data during ongoing spread [6], or has split data non-randomly into training and testing sets, either geographically [12,13] or based partially on chronology [5]. One study created chronological training and testing sets, to look at effects of climate change rather than to predict spread [14]. In general, a single pre-selected modelling method was used; only three of the aforementioned studies used multiple SDM methods [6,11,14]. No study to date has used chronologically subdivided data to predict species spread with multiple SDMs.

Our study extends these investigations by addressing how effectively different SDMs predict ongoing disease spread. For this purpose, non-equilibrium data from a still-spreading species are ideal. *Bd* is a massively destructive infectious fungus of amphibians that fits these criteria [15]; additionally, its time series of data is long enough to subset chronologically, such that model training data is temporally distinct from test data. We fit eight SDMs to discriminate presence from background (also known as ‘pseudo-absence’) data, to see how well incomplete, time-limited datasets predicted subsequent *Bd* records. Our aim was to assess the ability of different methods to forecast distributions of emerging diseases, not necessarily identify the best climatic model for *Bd* distribution. In the case of *Bd*, integrating existing mechanistic information might enable a more robust process-based model, but such models are often impractical in the broader context of emerging diseases because of their extensive data requirements. Statistical SDMs, despite their limitations, are therefore more appropriate for our purposes.

We hypothesized that prediction accuracy would improve for all models with a longer time series of training data [5]. We also predicted that boosted regression trees (BRT) and MaxEnt modelling methods would perform best, based on these methods' high levels of performance in previous papers ([16], Patel *et al.*, unpublished data) and the popularity of MaxEnt [17,18]. Largely in agreement with this prediction, we found that random forests (RF) and BRT best predicted the realized spread of *Bd—*closely followed by MaxEnt. We expect these modelling techniques to be useful for identifying and monitoring areas vulnerable to other emerging and non-equilibrium diseases.

## 2. Methods

**Figure 1.**
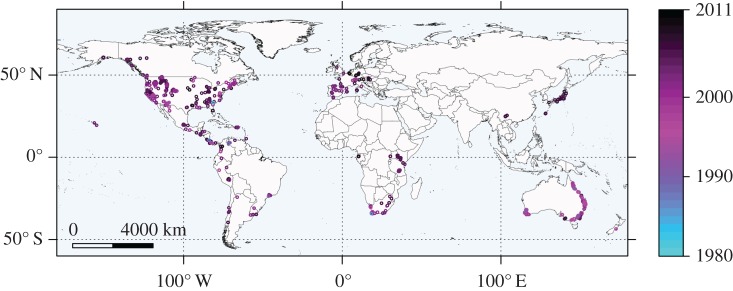
World map of *Bd*-positive sample locations used in this study. Points are coloured along a gradient by year of detection (1980–2011) and plotted in reverse chronological order. This ordering was chosen so that older occurrences would be visible in the clusters of *Bd* detections.

We obtained *Bd* occurrence data from the Global *Bd*-Mapping Project [15]. This dataset contains both positive and negative records from samples tested for *Bd*; samples were taken from living and dead animals [15]. We used only the positive records for our study because many datasets on spreading species do not contain accurate information about absences. In addition, because we were interested in predicting future spread, including absence data for *Bd* could have led to contradictions between training and test sets, for example, if a site was marked negative in one year and then found to be positive in a later year. Records prior to 1980 were removed because they were both sporadic and erratic; we also limited our consideration to sites with more than one *Bd*-positive individual to reduce false negatives in the modelling data. The final dataset we used for analysis contained 970 positive locations from 1980 to 2011 (figure 1). For statistical comparison, we sampled 20 000 background points (sometimes called ‘pseudo-absences’) from within 500 km buffers around the presence points; points that fell in the ocean were removed, leaving a total of 12 457 background points. Sampling background points from within buffers helps avoid excessively coarse-scale predictions [19]; because *Bd* has a widespread distribution and is effective at dispersing, we used relatively large 500 km buffers. We did not further limit buffer areas by host occurrence because amphibians occur widely (see fig. 1 in [20]) and *Bd* may survive outside of its usual host species [1,10,11]. We used sampled background points to fit and evaluate all models, even those that are traditionally presence–absence (as in [2,16,18]); presence-only models were trained only on presence points and then evaluated similarly with background points.

Rasters of 55 climate variables at 2.5 and 5 min resolution were obtained from the WorldClim database ([21]; see electronic supplementary material, table S1 for complete list of variables). These data include 19 bioclimatic variables, along with monthly layers for precipitation, minimum temperature and maximum temperature, representative of 1960–1990 average climate [21]. We note that these data, although widely used in species distribution modelling, do not account for climate change over the study period (1980–2011). Constructing similarly high resolution climate maps at a finer temporal resolution would be a massive undertaking, however, and beyond the scope of this project's resources or goals. The simplifications involved in using time-averaged climate data introduce inaccurate model predictions only to the extent that species distributions are in fact responsive to high frequency fluctuations rather than long-term averages. The (relatively high) measured accuracy across time periods is a partial assessment of model robustness to variations in climate.

Climate variables were rescaled and/or transformed to improve learning from highly skewed distributions (electronic supplementary material, table S1). Climate data for presence and background locations were extracted from the 2.5 min rasters, while global predictions with fit models used 5 min data to speed up computer processing. Recalling that our goals are partly methodological (to compare the accuracy of different SDM approaches under non-equilibrium conditions), we note that the most useful models for general application are those able to distinguish informative from uninformative variables [22,23]. While there is prior information about predictor variables relevant to *Bd* [11,15,24], this will not be the case for many newly emerging species, and was not the case throughout the spread of *Bd*. Thus, our study used all available covariates in recognition of intended future use of these methods. For similar reasons, we did not consider *Bd* niche shifts in our study, as including such complexities would require data likely to be unavailable for newly emerging diseases.

We evaluated eight models: boosted regression trees (BRT, presence-background; [25]), maximum entropy (MaxEnt, presence-background; [26]), logistic generalized linear modelling (GLM, presence-background; [27]), k-nearest neighbour (k-NN, presence-background; [28]), (RF, presence-background; [29]), plug and play Gaussian (PPG, presence-background; [30]), range bagging (RB, presence-only; [31]) and ecological niche factor analysis (ENFA, presence-only; [32]). Analysis was done in R v. 3.2.4 (RStudio; [33,34]), and the code is available with the data on Dryad (doi:10.5061/dryad.3p121).

**Figure 2.**
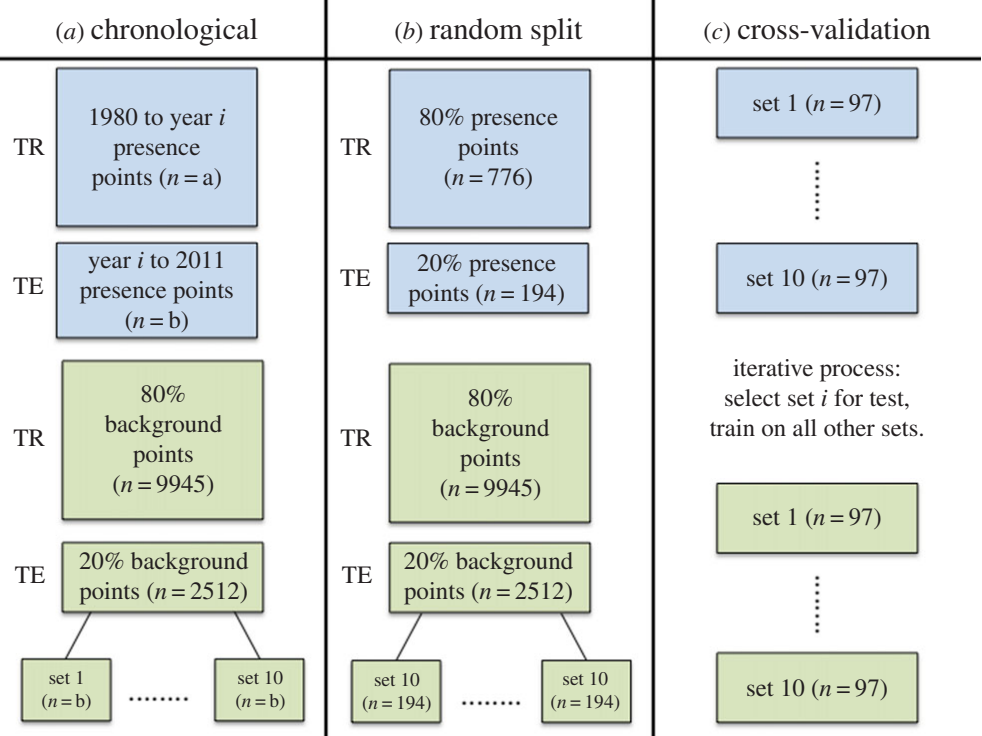
Flowchart of data divisions. Blue boxes represent presence points; green boxes represent background points. TR stands for training data and TE for test data.

We divided data into training and test sets in four ways, two of which were chronological (figure 2). For these chronological divisions, we trained models on a defined time period (1980 to year *i*) and tested on the remainder (year *i* + 1 to 2011; figure 2*a*). First, we used the period from 1980 to 1995 (24 presence points) and proceeded in 4-year time steps, ending up with four blocks to compare (*i* ∈ {1995, 1999, 2003, 2007}). An alternative chronological subsetting pattern redistributed this time-frame because years 2005 and 2006 contained 165 and 150 presence points, respectively, considerably more than most other years. In the first set-up, these two years fell into the same subset, resulting in their removal from testing data and addition to training data all at once. The alternative subset process split 2005 and 2006 into separate blocks, more evenly distributing the training and test points through time. Here, the first block was 1980–1996 (60 presence points), and blocks proceeded in 3-year time steps for five total (*i* ∈ {1996, 1999, 2002, 2005, 2008}). In each case, 9945 background points were used for training models that required background records. These two chronological analyses were then compared.

Our test sets contained many more background points than presence points, particularly in later time steps. To avoid inconsistent performance estimates from test datasets with excess background points, we tested models using balanced presence-background sets. At the beginning of each time step, 10 sets of background points were sampled from the 2512 background points withheld from training data; each of these 10 sets contained the same number of points as the current test set of presence points. Each model was then evaluated on the presence test set in conjunction with each background set to assess influence of variation in background point location.

In addition to comparing predictions from the two time-based splits, we ran the models on training and test sets created without respect to chronology, i.e. following the conventions most commonly adopted for assessing performance of SDMs [35]. We trained models on a random sample of 80% of the *Bd* data and tested on the remaining 20% (figure 2*b*). As before, 9945 background points were used for training models and tests were performed with balanced presence-background test sets. We also performed standard 10-fold cross-validation on all of our data, splitting our 970 presence points into 10 sets of 97 points (figure 2*c*). We created 10 sets of 97 pseudo-absence points for balanced test data, and created pseudo-absence training sets by removing one set of 97 points at a time from the full complement of 12 457 background points. For ENFA, we excluded one fold because it returned complex values in the ENFA model object. We included these randomized set-ups (i) to check that our environmental predictor variables could in fact predict *Bd*-positive areas, and (ii) to check for model selection agreement between time-dependent and standard assessments.

Performance of each model was evaluated using four different statistics. Area under the receiver operating characteristic curve (AUC; [36]) quantifies the performance of a binary classifier system and is the most commonly used evaluation for SDMs [37]. Receiver operating characteristic curves are constructed by varying the discrimination threshold and plotting true positive rate versus false positive rate at each threshold; the AUC is then the area under that curve. An AUC of 1 indicates a perfect direct relationship between prediction and truth (all true positives), and 0 indicates a perfect inverse relationship (all false positives). The AUC statistic may be interpreted probabilistically as the chance that a randomly chosen positive instance will be ranked higher than a randomly chosen background instance by the model [16,36].

The false negative rate measures how often a model misclassifies positive occurrences as negative. False negative rate ranges from 0 to 1. A value near 0 indicates that the model correctly identifies almost all presence points as presences; a value near 1 indicates that the model misidentifies almost all presence points as absences. False negative rate is not as widely reported as AUC for SDM evaluation, but it offers valuable insight here. We are concerned with where a disease will spread, to monitor and protect those areas; for this purpose, it is more important to account for all at-risk areas than to avoid false positives, which are equally weighted in the AUC.

Kappa [38] measures how closely model predictions match the truth, controlling for random accuracy by estimating the expected accuracy; it is a widely used evaluation measure for SDMs [39]. Kappa values range from −1 to 1, where 1 indicates 100% agreement, and −1 indicates 100% disagreement. For model selection, we referred to kappa secondarily because it did not offer much insight not already provided by the AUC and false negative rate. To calculate kappa and false negative rate, we used the evaluation threshold that maximized TPR + TNR (true positive rate + true negative rate) in calculating AUC [40].

Finally, the Boyce index [41,42] is used to assess how well presence locations in the test set match the model's overall predictions of suitable areas. This index takes values from −1 to 1, where negative values mean the model generally predicts unsuitable areas where presence points are found, and positive values mean the model generally predicts suitable areas where presence points are found. We used the default version of the Boyce index from the ‘ecospat’ package in R (continuous with class size 0.1; [43]). We could not calculate the Boyce index for k-NN because of the model's strictly binary response values.

Using four evaluation statistics offers a more in-depth understanding of model performance, because different statistics help uncover different inaccuracies and identify the strengths and weaknesses of the methods [2]. However, good performance on evaluation statistics, namely AUC, does not necessarily imply accurate geographical predictions [44]. Thus, a set of best models selected on the basis of evaluation statistics may not show spatial consensus. To explore this possibility, we made comparison maps of RF, BRT and MaxEnt (see Results). We rescaled map values so that in all cases the minimum predicted value was 0 and the maximum predicted value was 1. We computed the difference between rasters, with a difference near zero indicating similarity and values near 1 or −1 indicating disagreement among model predictions.

## 3. Results

**Figure 3.**
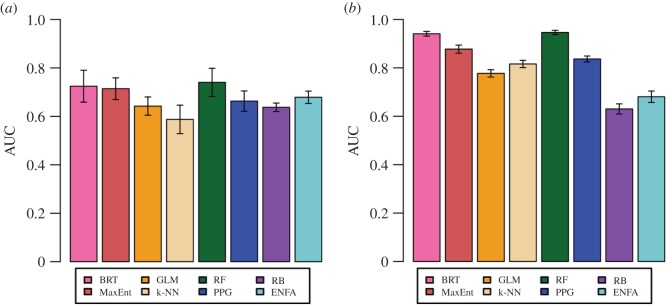
AUC values with 95% CIs, from (*a*) five-part chronological analysis, averaged over time steps, and (*b*) 10-fold cross-validation, averaged across folds.

RF, BRT and MaxEnt had the highest average AUC scores on chronological analyses, although their confidence intervals (CI) overlapped with some of the other methods (figure 3*a*). Average false negative rates for RF, BRT and MaxEnt did not stand out from the other methods (electronic supplementary material, figure S1*a*). In the random split and the 10-fold cross-validation, however, RF and BRT performed significantly better than the others (including MaxEnt) on AUC and kappa, with nearly indistinguishable evaluation statistics (figure 3*b*; electronic supplementary material, figure S2; kappa not shown). False negative rates for RF and BRT were among the lowest achieved, but not significantly so (electronic supplementary material, figure S1*b*,*c*). Based on these combined results, RF and BRT were the best models for predicting the spread of *Bd*, and MaxEnt also performed well (figure 3; electronic supplementary material, figures S1 and S2). On the chronological analyses, k-NN was the worst performing model; GLM was worst on the random split, followed by RB, both of which also performed poorly in 10-fold cross-validation.

**Figure 4.**
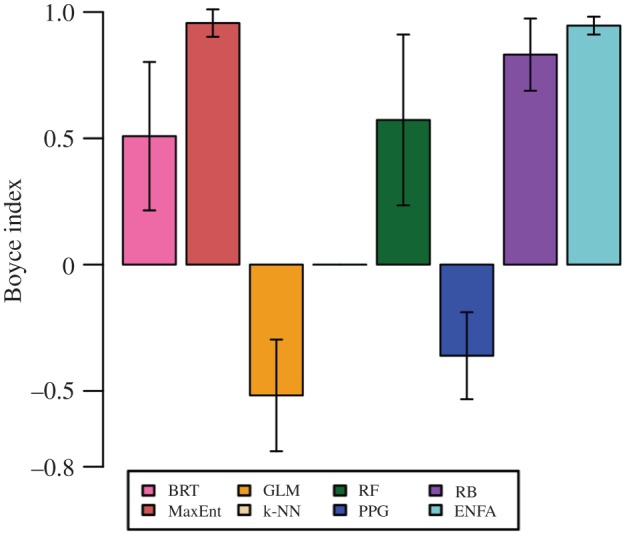
Boyce index values, with 95% CIs, from five-part chronological analysis, averaged over time steps.

**Figure 5.**
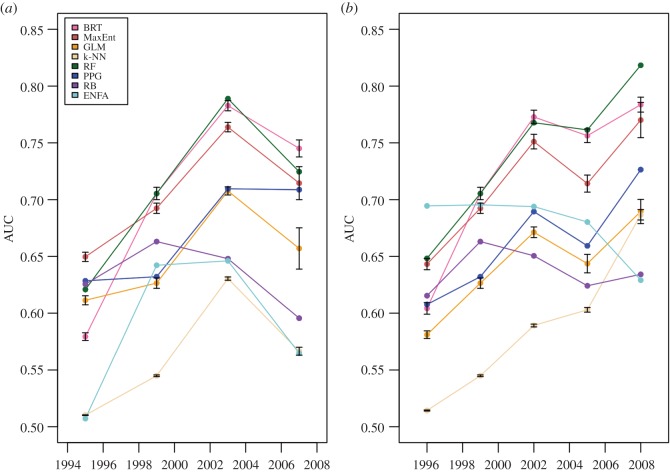
(*a*) AUC values for all models from the four-part chronological analysis. Seven of the eight models see their AUCs drop from the third to fourth time step. (*b*) AUC values for all models from the five-part chronological analysis. Six of the eight models reach their highest AUC at the fifth and final time step. Error bars represent 95% CIs, from evaluating models on 10 different sets of background data, each in conjunction with the test set of presence points.

The Boyce index (figure 4) was highly variable and uncorrelated with AUC values. We noticed this contradiction most strikingly in the five-part chronological analysis for RF and BRT. Boyce index values for both models dropped sharply at the final time step (electronic supplementary material, figure S3), while AUC values increased (figure 5*b*). Overall, we saw poorest performance with respect to the Boyce index for models that are not usually run in presence-only contexts (e.g. GLM and PPG; figure 4).

The three models that performed best on average—RF, BRT and MaxEnt—were also significantly better than the others at almost every time step (figure 5). In the four-part chronological analysis, we expected the initial upward trend in AUC scores, as observed, as a longer time series of training data allows for more refined prediction of future detection areas (figure 5*a*). However, we also observed an unexpected decline at the final time step (discussed further below). In the five-part chronological analysis, this drop in AUC values was reduced and shifted. The performance of several models dipped slightly at the penultimate time step, when 2005 was switched from the testing to training data. We nevertheless found most models to achieve their highest AUC score on the last time step (figure 5*b*). Thus, our results generally supported the hypothesis that the accuracy of predictions improves with the length of time used for training, but we also found that actual patterns in spread or sampling affect those increases in model performance.

**Figure 6.**
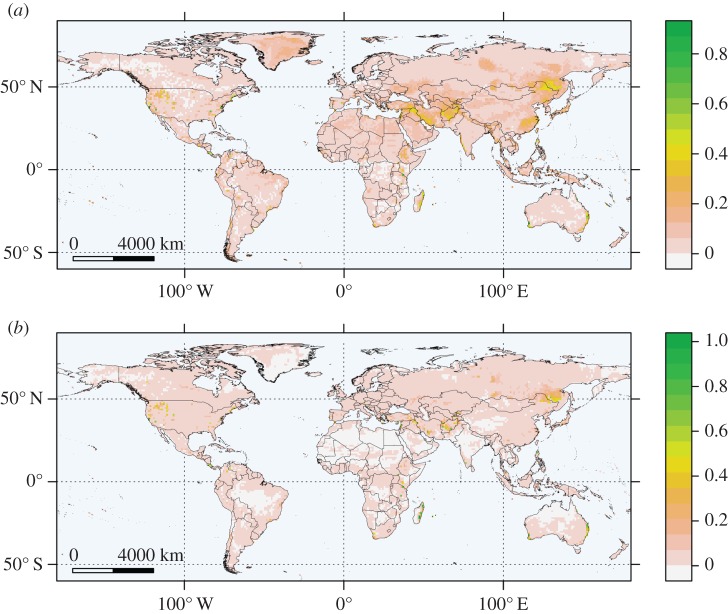
(*a*) Random forest and (*b*) boosted regression trees prediction maps from the five-part chronological analysis. Training data were *Bd* points from 1980–2008; test data were points from 2009–2011.

We used maps of suitability to visually compare outputs of the two best models, RF and BRT. Mapped predictions (figure 6) generally aligned with *Bd* observations (figure 1) and are similar between the two models. Model outputs suggested that Southeast Asia and Australia, already infected by *Bd*, could see further interior spread (figure 6). Additionally, both models identify suitable uncolonized areas in eastern Madagascar, the Middle East and near the Chinese-Russian border (figure 6).

**Figure 7.**
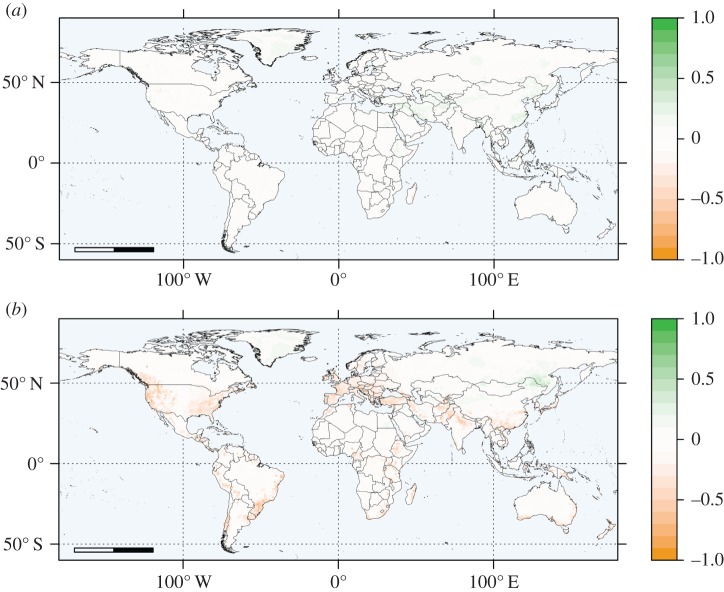
Comparison maps for the three best performing models, RF, BRT and MaxEnt, based on final predictions from the five-part chronological analysis. Green represents areas predicted to be more suitable by RF than by the other model. Orange represents areas predicted to be more suitable by the other model than by RF. (*a*) RF versus BRT. Raster values for map calculated as RF prediction value minus BRT prediction value. (*b*) RF versus MaxEnt. Raster values for map calculated as RF prediction value minus MaxEnt prediction value. BRT versus MaxEnt comparison map (electronic supplementary material, figure S4) is nearly identical to (*b*).

In summary, quantitative comparison confirmed high spatial consensus among our top methods (RF, BRT and MaxEnt; figure 7). As all three models were fit with background data, cell values in their predictions represented relative risks, not probabilities [3,16]. The spatial extents of these risks initially had different numerical ranges, so we rescaled prediction values to facilitate comparison of the most and least suitable areas identified by each model. Visual inspection shows that RF and BRT were more similar to each other (figure 7*a*) than to MaxEnt (figure 7*b*; electronic supplementary material, figure S4). Despite small differences, the three methods generally agreed, while poor performing methods such as RB showed little agreement with either RF or BRT (electronic supplementary material, figure S5). We cannot make an inductive statistical claim based on these results, but the agreement among the top models is encouraging and our case study provides guidance for future analysis.

## 4. Discussion

Overall, performance of models fit to chronologically arranged data was high enough to suggest that useful results can be extracted from the early stages of spread (top three models, average AUC across time steps: 0.71–0.74), but was still substantially lower than cross-validation on the full dataset (top three models, average AUC across 10 folds: 0.88–0.95). This difference quantifies the magnitude of the ‘non-equilibrium problem’ in modelling disease emergence, but also demonstrates that SDMs may nevertheless be used for predicting spread. Our approach (incrementing training and test data by time) assessed equilibrium assumptions in a way that randomized splitting cannot, but in this particular case, both set-ups led us to select the same models, despite numerical differences in performance. Additionally, the best models for predicting the past spread of *Bd* (RF, BRT and MaxEnt) were the same methods found to excel in other species distribution contests (e.g. [2,16]). As researchers working with newly emerging diseases may not have enough data to subset the occurrences by time, this intra- and inter-study agreement about best models is encouraging, particularly given the practicality and widespread use of randomized splits for SDM evaluation.

It is also important to note that model performance depended on how many years of data were available for model training. For example, PPG and RB had similar AUC values on initial time steps, but by the final time step they were significantly different (figure 5). The overall best models (RF, BRT and MaxEnt) would not be easily identified as such from only the first time steps (figure 5). Variation in chronological data division also influenced model performance. Particularly, we found ENFA to be highly inconsistent. Its lowest AUC score from the five-part chronological analysis (figure 5*b*, 2008) is just below its highest score from the four-part chronological analysis (figure 5*a*, 2003). With a limited time series or single pattern of chronological division, then, results are expected to be highly uncertain. Additionally, averaging across time steps (figure 3*a*) obscured significant differences among models (figure 5), leading us to conclude that it is important to look at model performance at multiple time points, rather than in a single snapshot.

In assessing model performance, we noticed that evaluation statistics for the three best models were fairly similar overall. RF and BRT are both ensembles of trees, while MaxEnt is a regression algorithm [18,23]. RF and BRT are highly effective in fitting SDMs because they allow the most flexibility in modelling nonlinearities and interactions, and can handle correlated predictors [23,45]. The next best model, MaxEnt, is also designed to handle interactions and nonlinearities, such as thresholds [26], emphasizing the importance of this flexibility in predicting future distributions. Interestingly, Holmes *et al.* [18] selected RF, BRT and MaxEnt for an ensemble modelling project because the three methods complement each other in how they use data and in the assumptions they make. Not only do Holmes *et al.* [18] set a precedent for consensus modelling with these methods, but as each method performs well on its own, they ought to do well together too. Such an approach is similar to using more than one evaluation statistic for a more thorough model selection process, in that it too can help emphasize particularly good predictions (high consensus among models) while also indicating which predictions are more uncertain (low consensus among models; [1]). Our results show that some methods may need to be avoided in creating an ensemble of models, as they were unable to effectively model the ongoing spread.

We did not observe a steady increase in model performance over time (figure 5), for which there are multiple potential causes. Spatial autocorrelation could have contributed to the erratic evaluation patterns, as data subsets constructed from incremental time intervals tend to include spatially clustered points. However, we did not want to explicitly control for such clustering because it follows how actual detection of spread occurs, and we were interested in predicting that spread. For the four-part chronological analysis in particular, the non-monotonic pattern of AUC values (figure 5*a*) could be due to the abrupt loss of points from the test set at the fourth step, when years 2005 (165 presence points) and 2006 (150 presence points) shifted from training to testing data. Splitting the data into smaller time steps gave us better overall model performance and led to results (e.g. patterns in AUC values) that better fit expectations (figure 5*b*). Smaller increments of data allow models to incorporate more nuances in sampling and spread over time; the training and test sets shift more gradually, so it makes sense that we would generally see less dramatic jumps in AUC from one time step to the next. For species with sufficient data, researchers might consider running models with 1-year time steps. Such a set-up could offer additional information on how sampling efforts affect predictions—for example, whether variation in the number of new presence points found each year strongly influences predictions. One might also examine year-to-year changes in the geographical area and environmental space predicted suitable for a species. When these stop changing, meaning further sampling efforts no longer provide new information, the species has probably reached environmental equilibrium. Geographical spread may still occur, but it will be within the known environmental space.

James *et al.* [11] proposed looking for *Bd* ‘cold spots’ to better understand its limiting factors, but they did not specify how researchers might recognize such locations from modelling results. We suggest AUC and false negative rate, taken together, as useful evaluation statistics for identifying where the pathogen cannot or will not establish. AUC values alone, without any corroborating statistic, could be misleading in identifying accurate cold spot predictions, because models can get reasonable AUC scores just by predicting core habitat areas. Identifying *Bd*'s core habitat constitutes better-than-random prediction (AUC > 0.5); however, areas determined to be unsuitable then represent both true cold spots and an unknown degree of model underprediction. False negative rate could provide an estimate of this underprediction; one could assess relative confidence in cold spot predictions by comparing false negative rates of models with similar AUC values. The best model would maximize AUC while minimizing false negative rate. Thus, the two statistics could be used in conjunction to assess the reliability of a model's cold spot detection.

Process-based models have been argued as more appropriate for extrapolation than the correlative SDM techniques used here [46,47], but the intense data and information requirements for process-based models generally render them impractical in the context of newly emerging diseases. We believe SDMs are therefore a good option for this purpose, while having some limitations. For example, distinguishing causal relationships from correlations between climate variables and species range limits is not possible, as observed presences are the result of complex interactions between factors both enabling and limiting species occurrence [46,47]. Furthermore, there is considerable debate and uncertainty as to which aspect(s) of a species' niche these models capture [48–50]. We used SDMs not because they are the most realistic models, but because they allow us to make projections with the limited data most often available. It would be interesting to compare the results of a process-based *Bd* model with our SDM results, to better understand the effort involved and whether important differences are revealed.

In general, AUC values from different studies cannot be naively compared [4,6]. The selection of background data contributes strongly to this. Extreme geographical or environmental distance between background and presence points can artificially inflate AUC [19]. Additionally, using background data for model evaluation lowers the maximum attainable AUC value from 1 to 1 − *a*/2, where *a* is the (unknown) prevalence of the species on the landscape [26,51]. This means that, even apart from any bias due to selection of background data, our AUC values are on a slightly different scale than the usual 0 to 1 range. However, multiple other *Bd* modelling investigations have also used background data for model evaluation, and while this does not resolve the bias or imply that our AUC scores are on exactly the same scale, a rough comparison might be warranted. Randomized split AUC scores for our top three models (0.88–0.95) are generally consistent with those found in other studies (0.91 [8]; 0.90 [11]; 0.79–0.99 [18]; 0.81–0.84 [52]), showing that our models compare favourably to those used in other studies.

Previous work has found that AUC is strongly correlated with the continuous Boyce index (Pearson's correlation coefficient = 0.8; [42]). By contrast, our data showed a much weaker correlation (Pearson's correlation coefficient = 0.255, *p* < 0.001). This could be because the Boyce index is a presence-only evaluation, and many of our models are presence–absence methods. Given increasing use of the Boyce index for SDM evaluation (e.g. [53–55]), this discrepancy warrants further investigation.

## 5. Conclusion

In conclusion, we found that RF and BRT, followed by MaxEnt, best predicted subsequent *Bd* records from time-limited subsets of data, and that these models performed much better than random in identifying the future range of the pathogen even with small amounts of data. While we would urge that any model results be interpreted cautiously and with respect to a specific question and data [2,3], these results suggest that RF, BRT and MaxEnt are good choices for modelling emerging and non-equilibrium species.

## Supplementary Material

Supplementary material: Accuracy of climate-based forecasts of pathogen spread

## Data accessibility

All code and data are available on Dryad (http://dx.doi.org/10.5061/dryad.3p121) [56].
