# Supplementary material for: Accuracy of climate-based forecasts of pathogen spread
Source: R Soc Open Sci. 2017 Mar 29;4(3):160975. doi: 10.1098/rsos.160975 (PMC5383844; doi:10.1098/rsos.160975)
Supplement: Supplementary material: Accuracy of climate-based forecasts of pathogen spread [file rsos160975supp1.pdf]

Accuracy of climate-based forecasts of pathogen spread

SUPPLEMENT

Annakate M. Schatz, Andrew M. Kramer, and John M. Drake

Royal Society Open Science

Methods: Model Settings

All model code can be found on Dryad (doi: 10.5061/dryad.3p121).

Boosted Regression Trees: To fit the model, we used the "Bernoulli" family with an individual tree complexity of 5, a learning rate of 0.01, and a bag fraction of 0.5. To predict, we used 'best.trees' for the number of trees.

- R packages: 'dismo' (1) for model fitting; 'gbm' (2) for predicting.

Maximum Entropy: Our predictor input was a RasterBrick of climate data.

- R packages: 'dismo' (1) package for model fitting; also requires 'rJava' (3).

Generalized Linear Modeling, logistic: We used the binomial family with the logit link function.

- R packages: 'stats' (4) for model fitting.

k-Nearest Neighbor: We set the number of neighbors to k=3.

- R packages: 'FNN' (5) for model fitting.

Random Forest: We ran this model as regression.

- R packages: 'randomForest' (6) for fitting

Plug and Play Gaussian: Code was provided by JMD. We used the "regularized" method.

Range Bagging: Code was provided by JMD. We set this model to use 256 votes and bag 1 dimension of ranges, with proportion of points used set to 0.8.

Ecological Niche Factor Analysis: We converted the pixel utilization weights to 0s and 1s, to be consistent with the other models. To evaluate and map model predictions, we first transformed the ENFA prediction object to a raster and standardized using an ecdf transformation. Then we subtracted that result from 1 for the final raster to evaluate and map.

- R packages: 'adehabitatHS' (7) for model fitting and prediction; 'sp' (8,9) for preparing presence data for model input.

Additional R package notes: 'dismo' (1) for evaluation, prediction, and pseudo-absence buffers; 'maptools' (10) for world map outline; 'raster' (11) for prediction; 'rgdal' (12) for setting CRS of climate layers and creating pseudo-absence buffers; 'rgeos' (13) for creating pseudo-absence buffers; 'sp' (8,9) for sampling pseudo-absence points.

References

1. Hijmans RJ, Phillips S, Leathwick J, Elith J. dismo: Species distribution modeling. 2013. R package version 0.8-11. https://CRAN.R-project.org/package=dismo.

2. Ridgeway G, with contributions from others. gbm: Generalized Boosted Regression Models. 2013. R package version 2.1. https://CRAN.R-project.org/package=gbm.
3. Urbanek S. rJava: Low-level R to Java interface. 2013. R package version 0.9-4. https://CRAN.R-project.org/package=rJava.
4. R Development Core Team. R: A language and environment for statistical computing. Vienna, Austria: R Foundation for Statistical Computing; 2008. http://www.r-project.org.
5. Beygelzimer A, Kakadet S, Langford J, Arya S, Mount D, Li S. FNN: Fast Nearest Neighbor Search Algorithms and Applications. 2013. R package version 1.1. https://cran.r-project.org/package=FNN.
6. Liaw A, Wiener M. Classification and Regression by randomForest. 2002. R News 2(3): 18-22. http://cran.r-project.org/doc/Rnews/.
7. Calenge C. The package adehabitat for the R software: a tool for the analysis of space and habitat use by animals. 2006. Ecological Modelling 197: 516-519.
8. Pebesma EJ, Bivand RS. Classes and methods for spatial data in R. 2005. R News 5(2): 9-13. http://cran.r-project.org/doc/Rnews/.
9. Bivand RS, Pebesma EJ, Gomez-Rubio V. Applied spatial data analysis with R, 2nd edition. 2013. Springer, NY. http://www.asdar-book.org/.
10. Bivand RS, Lewin-Koh N. maptools: Tools for reading and handling spatial objects. 2013. R package version 0.8-24. https://CRAN.R-project.org/package=maptools.
11. Hijmans RJ. raster: Geographic Data Analysis and Modeling. 2015. R package version 2.4-20. https://CRAN.R-project.org/package=raster.
12. Bivand RS, Keitt T, Rowlingson B. rgdal: Bindings for the Geospatial Data Abstraction Library. 2015. R package version 1.0-5. https://CRAN.R-project.org/package=rgdal.
13. Bivand RS, Rundel C. rgeos: Interface to Geometry Engine - Open Source (GEOS). 2013. R package version 0.2-19. https://CRAN.R-project.org/package=rgeos.

Methods: Climate Variables

Table S1. Fifty-five climatic variables used in model fitting. The empirical cumulative distribution function (ecdf) transformation was used when log transformation failed to reduce skewness and outliers in the data. ecdf creates a uniform distribution, and was computed on values in a raster and applied to that raster to return a rescaled version. After transformations listed in table, all variables (excluding ecdf variables) were rescaled by subtracting mean and dividing by standard deviation. Units for 'tmin' and 'tmax' are ºC * 10. Units for 'prec' are millimeters. For units of bioclimatic variables, see http://worldclim.org/bioclim.

Variables	Information	Mean (2.5-min, 5 min)	Standard Deviation (2.5-min, 5 min)	Transformations
Bio1	Annual Mean Temperature	81.61, 81.42	147.96, 148.18	
Bio2	Mean Diurnal Range	111.37, 110.89	31.22, 31.34	
Bio3	Isothermality	38.19, 38.19	20.62, 20.67	
Bio4	Temperature Seasonality	8302.76, 8282.27	5200.44, 5200.29	
Bio5	Max Temp. of Warmest Month	258.15, 257.45	103.15, 103.30	
Bio6	Min Temp. of Coldest Month	-90.45, -90.04	199.56, 199.8	
Bio7	Temperature Annual Range	348.60, 347.49	137.81, 138.08	
Bio8	Mean Temp. of Wettest Quarter	152.70, 152.32	105.67, 105.75	
Bio9	Mean Temp. of Driest Quarter	23.67, 23.70	204.73, 204.95	
Bio10	Mean Temp. of Warmest Quarter	186.05, 185.64	96.46, 96.61	
Bio11	Mean Temp. of Coldest Quarter	-25.36, -25.22	203.79, 203.99	
Bio12	Annual Precipitation	705.24, 709.23	680.97, 686.58	ecdf
Bio13	Precipitation of Wettest Month	116.86, 117.31	111.41, 112.19	ecdf
Bio14	Precipitation of Driest Month	20.86, 21.10	31.36, 31.71	ecdf
Bio15	Precipitation Seasonality	60.48, 60.32	33.93, 33.87	
Bio16	Precipitation of Wettest Quarter	309.36, 310.63	296.15, 298.32	ecdf
Bio17	Precipitation of Driest Quarter	73.36, 74.11	104.23, 105.38	ecdf

Bio18	Precipitation of Warmest Quarter	209.53, 210.19	187.72, 188.69	log
Bio19	Precipitation of Coldest Quarter	128.13, 129.35	206.7, 207.64	log
tmin1	Average Jan. min temperature	-72.86, -72.49	214.19, 214.37	
tmin2	Average Feb. min temperature	-63.01, -62.78	211.70, 211.93	
tmin3	Average Mar. min temperature	-30.53, -30.55	194.48, 194.86	
tmin4	Average Apr. min temperature	19.02, 18.82	158.36, 158.85	
tmin5	Average May min temperature	67.98, 67.83	116.83, 117.17	
tmin6	Average June min temperature	103.99, 103.90	91.2, 91.57	
tmin7	Average July min temperature	121.78, 121.72	82.91, 83.00	
tmin8	Average Aug. min temperature	114.65, 114.65	85.40, 85.51	
tmin9	Average Sept. min temperature	85.97, 86.01	101.52, 101.70	
tmin10	Average Oct. min temperature	40.09, 40.15	134.52, 134.80	
tmin11	Average Nov. min temperature	-17.11, -16.89	177.67, 177.88	
tmin12	Average Dec. min temperature	-55.96, -55.63	201.46, 201.62	
tmax1	Average Jan. max temperature	33.66, 33.63	230.24, 230.19	
tmax2	Average Feb. max temperature	48.69, 48.47	225.26, 225.34	
tmax3	Average Mar. max temperature	87.02, 86.51	203.75, 204.05	
tmax4	Average Apr. max temperature	137.41, 136.73	170.40, 170.78	
tmax5	Average May max temperature	183.65, 182.95	135.53, 135.82	
tmax6	Average June max temperature	219.89, 219.22	109.13, 109.32	
tmax7	Average July max temperature	236.73, 236.13	96.27, 96.40	
tmax8	Average Aug. max temperature	228.14, 227.59	100.86, 101.00	
tmax9	Average Sept. max temperature	195.49, 294.99	123.22, 123.36	
tmax10	Average Oct. max temperature	145.41, 145.02	159.48, 159.57	

tmax11	Average Nov. max temperature	86.16, 85.99	198.91, 198.91	
tmax12	Average Dec. max temperature	47.63, 47.58	220.08, 220.02	
prec1	Average Jan. precipitation	54.29, 54.70	78.05, 78.55	log
prec2	Average Feb. precipitation	50.13, 50.45	71.94, 72.31	log
prec3	Average Mar. precipitation	54.57, 54.86	75.19, 75.52	log
prec4	Average Apr. precipitation	52.75, 52.99	67.67, 67.91	log
prec5	Average May precipitation	56.36, 56.65	70.06, 70.43	log
prec6	Average June precipitation	62.95, 63.26	78.32, 79.00	log
prec7	Average July precipitation	73.04, 73.29	89.43, 90.00	log
prec8	Average Aug. precipitation	72.54, 72.80	83.54, 84.09	log
prec9	Average Sept. precipitation	63.89, 64.25	71.47, 72.01	log
prec10	Average Oct. precipitation	57.43, 57.85	65.51, 66.13	log
prec11	Average Nov. precipitation	53.32, 53.73	65.16, 65.71	log
prec12	Average Dec. precipitation	53.96, 54.38	73.24, 73.74	log

Figures

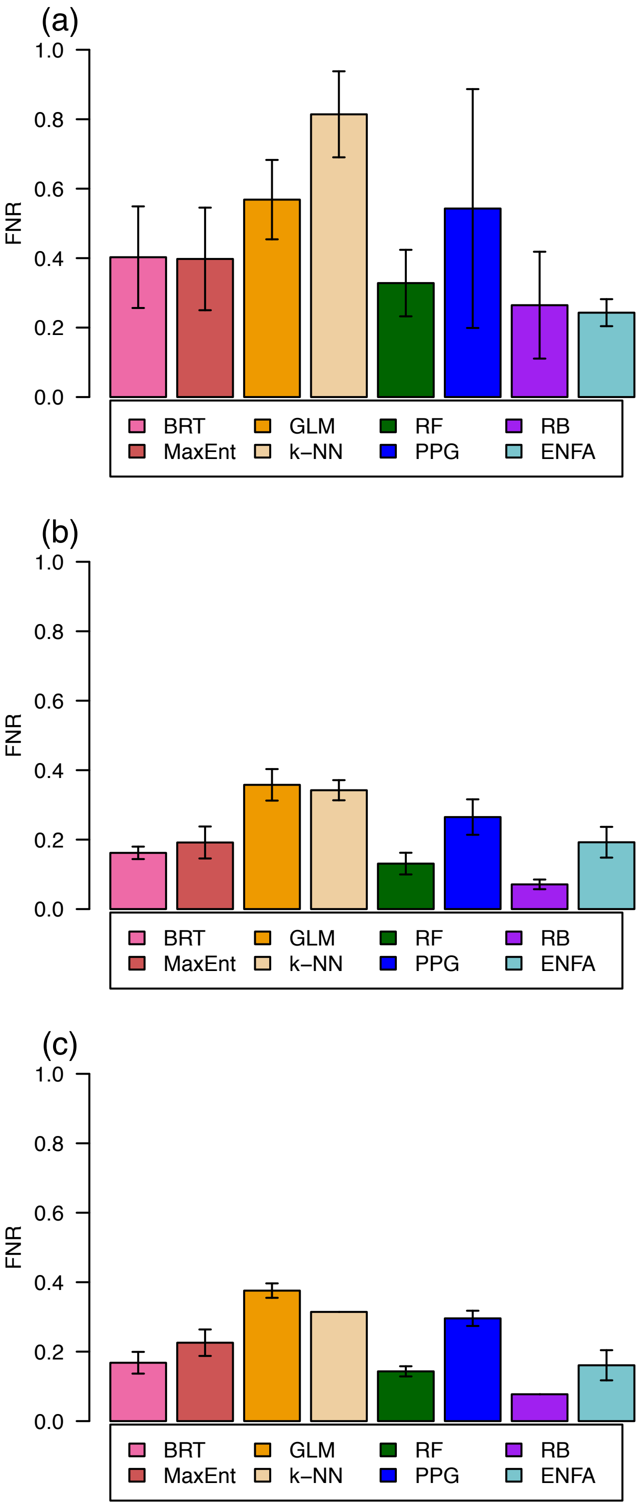

Figure S1. False negative rates, with 95% confidence intervals, for **(a)** five-part chronological analysis, averaged over time steps, **(b)** ten-fold cross-validation, averaged across folds, and **(c)** random split, averaged across balanced presence-background test sets.

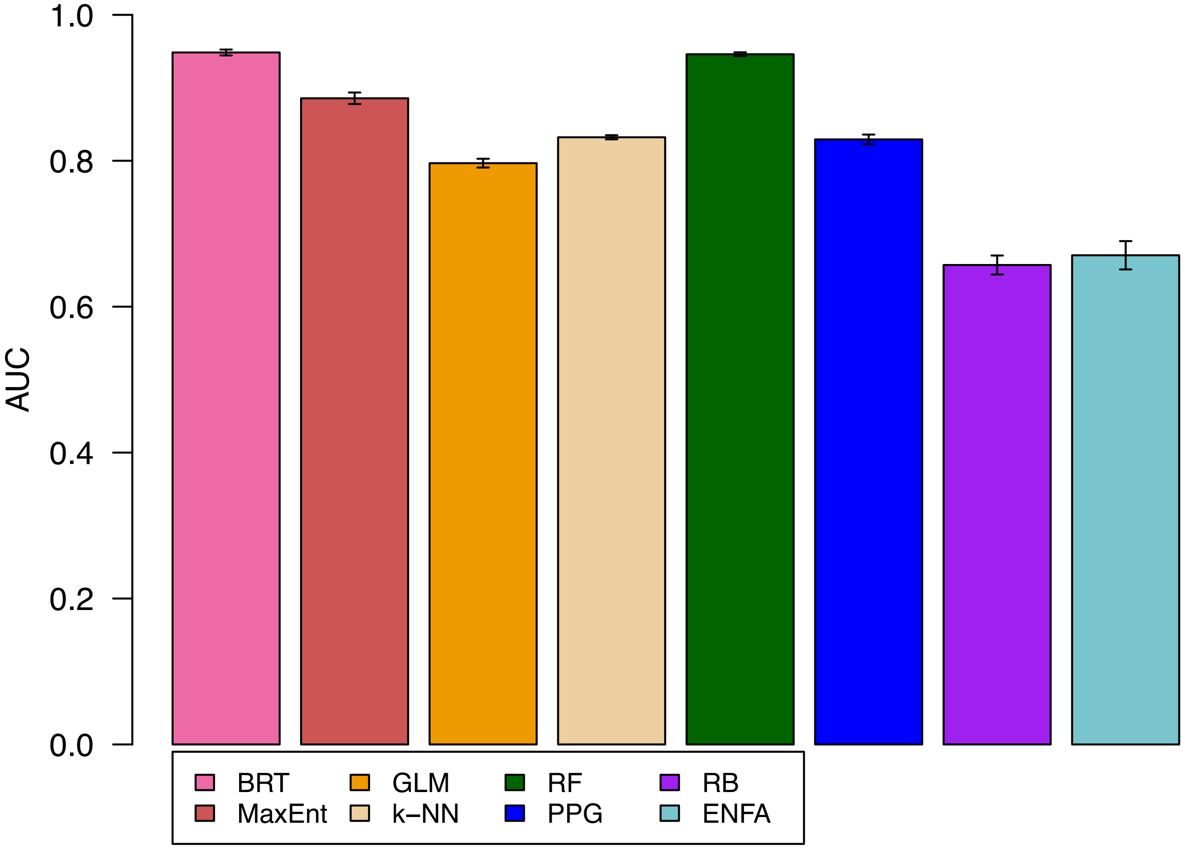

Figure S2. AUC values from the random split. Error bars represent 95% confidence intervals, from evaluating models on ten different sets of background data, each in conjunction with the test set of presence points.

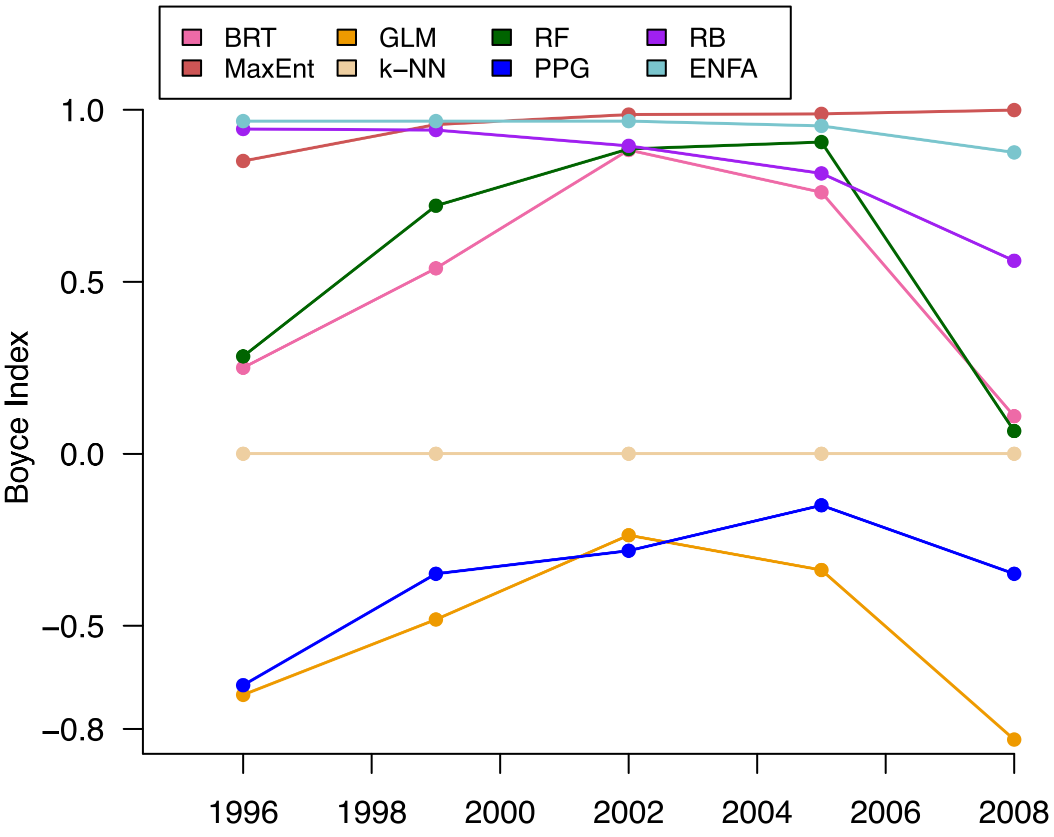

Fig. S3. Boyce index values from the five-part chronological analysis.

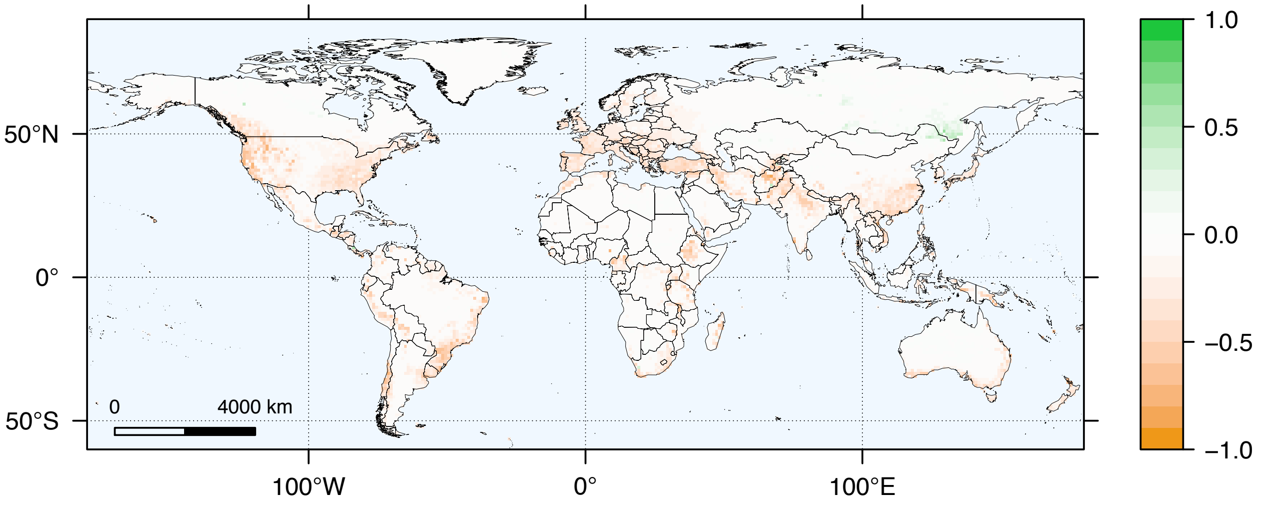

Figure S4. Comparison map for BRT vs. MaxEnt. Green represents areas predicted to be more suitable by BRT than by MaxEnt. Orange represents areas predicted to be more suitable by MaxEnt than by BRT. Raster values for map calculated as BRT prediction value minus MaxEnt prediction value.

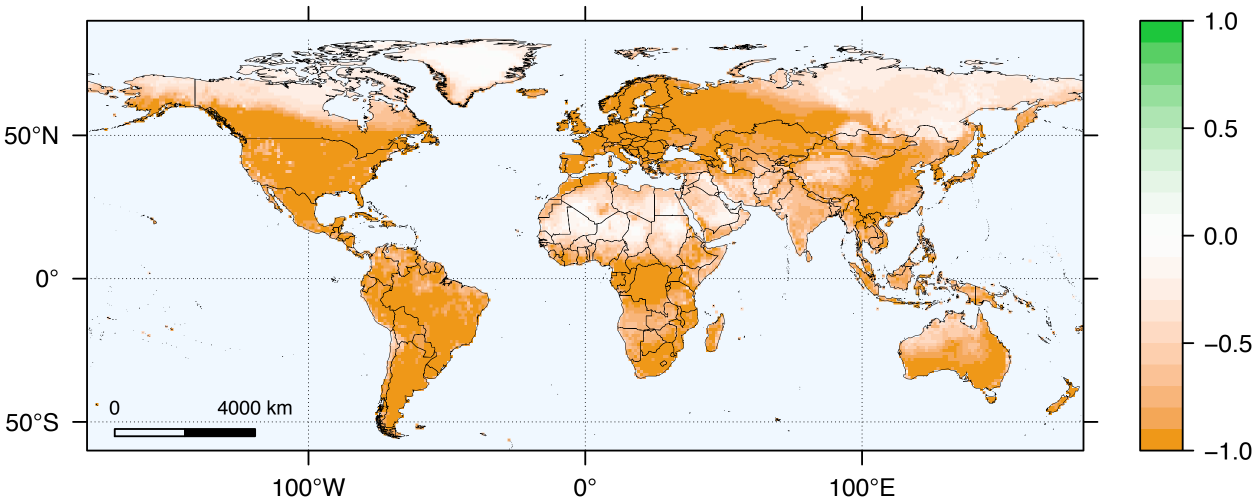

Figure S5. Comparison map for RF vs. RB. Green represents areas predicted to be more suitable by RF than by RB. Orange represents areas predicted to be more suitable by RB than by RF. Raster values for map calculated as RF prediction value minus RB prediction value. BRT vs. RB comparison map is nearly identical.
